# Items of General Interest

**Published:** 1915-09

**Authors:** 


### Items of General Interest.

---

Petitions are being signed asking the commissioners court of Hall county to order an election for the purpose of bonding the county in the sum of $30,000 for the erection of a county hospital.

---

El Paso will soon have a vote on a $100,000 bond issue for the establishment of a poor farm and the improvement of the county hospital.

---

Dr. Joseph Davis, accompanied by A. Schiafli, sanitary engineer, and a corps of assistants, have been in Orange conducting a sanitary campaign under the auspices of the State Health Department. While they were there the commissioners court authorized the payment of 5 cents bounty for every rat delivered to Dr. Davis during his two or three weeks' stay in the city. The city fully co-operated with the health department in the campaign.

---

Physicians in San Antonio will have green crosses painted on their automobiles hereafter instead of the ordinary red cross. An ordinance of that city requires them to have red crosses, but the Federal law prohibits to all but authorized persons the use of the red cross, which is the emblem of the American Red Cross Society. The green cross is the emblem of the American Medical Society and members of the local doctors' association have a right to use it. The local ordinance requiring a red cross is held to be void because of the prohibitive Federal law.

John Sealy contemplates the expenditure of $80,000 in the near future on improvements and additions for the John Sealy Hospital, which operates in Galveston in conjunction with the State medical college. It is understood that the changes include an additional story for each wing of the building and considerable enlargement of the clinical facilities. Plans are being drawn for the alterations. It will be remembered that Mr. Sealy has already spent thousands of dollars recently for improvements in the department of obstetrics. Although a charity hospital, no pains or expenses have been spared to make it one of the most modern hospitals in the United States.

The members of the Women's Christian Temperance Union filled the book cases recently donated to the Fort Worth City-County Hospital. Each member brought as many old books and magazines as she care to, and the affair was a tremendous success. The W. C. T. U. of that city has done much to help the inmates of the hospital recently.

---

A physician is the inventor of a hollow cane on which he carries all the medicine bottles he ordinarily needs to have with him.

---

One of the features for the benefit of the Civic League at Denison recently was a doctors-lawyers ball game, in which some of the most prominent men in town participated.

---

What are described as remarkable cures of wounded French soldiers have been effected by the new polyvalent serum, discovery of which was announced last March. Complete recovery is announced of men who were terribly mutilated and for whom all hope had been given up before use of the serum, so badly infected were their wounds.

Drs. Leclainche and Vallee, discoverers of the serum, have been unable up to the present to make more than 2000 flasks of it daily, most of which goes to the base hospitals, where the worst cases are to be found. When it can be made in sufficient quantities to supply the firing line, where it could be used preventively as an antitetanus serum is now used, it is believed that thousands of lives can be saved.

The new serum, which may be roughly described as a combination of a number of serums against different varieties of bacteria, has been put to practical use only recently.

An addition, to cost $200,000, has been signed by representatives of St. Paul's Sanitarium, Dallas, with H. J. Curtis, a contractor of that city. It will be in the nature of an east wing, and will almost double the present capacity of the institution.

---

At a Chicago medical congress cottage cheese was boosted as an unusual life food and nerve tonic. Dr. Gertrude Meek, of Cleveland, said the cheese contained much phosphorus. Elderly person were warned to eat less if they would live longer and more comfortably. Warning against the newly discovered serum treatment of rheumatism was issued by Dr. Frank Branen, of Chicago. Alcohol, tobacco and coffee, when used immoderately, produce palpitation of the heart, which some physicians mistake for heart disease, and thus worry some patients to death, said Dr. John Prentice Rand, of Worcester, Massachusetts. If you want to become fat, drink plenty of pure water, is the advice given by Dr. A. E. Smith, of Freeport, Illinois.

---

Physicians from several of the leading hospitals of New York City discussed with interest at a recent meeting the discovery of what was described as a harmless X-ray. Fourteen physicians had witnessed a demonstration of the new ray by Charles Stanley, an electrician, who claims to have discovered the ray, and who asserts it eliminates danger heretofore said to surround the application of the X-ray.

---

Miss Grace Gassette, an American portrait artist, has been placed at the head of the bandage department of the American ambulance hospital at Neuilly, where 7000 compresses, 2000 cotton balls, 400 rolled bandages, 300 combines and 1000 fluffs must be turned out daily. Miss Gassette has invented many devices which have aided in the caring for wounded. One of these is a wooden frame with a sliding cradle inside called "the leg puller," which prevents the bone from knitting wrong. The "field splint" and an anklet for low fractures are other devices of her invention.

---

The danger of the transmission of disease in swimming pools is becoming realized more prominently with the growing popularity of these institutions. Calcium hypochlorite has been highly recommended for disinfecting, and has been adopted for many pools. Its potential efficiency as a disinfectant for the water is not doubted. The use of the hypochlorite, however, is frequently accompanied by complaints regarding the disagreeable odor attending it. This fact had led to much experimentation in search of an equally efficient and inoffensive substitute. The difficulties here referred to are decidedly greater in connection with swimming pools than with public water supplies. For the treatment of the latter, more than two parts per million of hypochlorite are necessary. Even with this small proportion, aeration of drinking water is necessary to overcome the objectionable taste and odor. In the case of the swimming pool, this feature is aggravated by the fact that, according to trustworthy bacteriologists, three parts of the hypochlorite per million are necessary to sterilize the water. In tests conducted recently at the Taylor gymnasium pool at Lehigh University, South Bethlehem, Pa., S. T. Thomas has obtained encouraging results by the employment of copper sulphate in place of calcium hypochlorite. The possibilities of the application of copper sulphate as a disinfectant in polluted water is not new. The results recorded by Thomas are sufficiently promising to deserve further consideration by hygienists.

